# Perceptions of Mental Health Clinicians Working in Long-Term Care Facilities During the COVID-19 Pandemic

**DOI:** 10.1093/geroni/igab046.772

**Published:** 2021-12-17

**Authors:** Roma Hanks, Lisa Brown, Rachel Ward, Savannah Rose, Lisa Lind

**Affiliations:** 1 University of South Alabama, Mobile, Alabama, United States; 2 Palo Alto University, Moss Beach, California, United States; 3 Palo Alto University, Palo Alto, California, United States; 4 Deer Oaks Behavioral Health, San Antonio, Texas, United States

## Abstract

During the COVID-19 pandemic, mental health clinicians were initially not considered essential workers, and most were prevented from entering long-term care (LTC) facilities. This study investigated the perceptions and experiences of licensed clinicians who were providing services in LTC settings before and during the pandemic. Respondents included 126 clinicians from 31 states who completed a 90-item survey to assess the impact of COVID-19. Visitor restrictions were perceived to have had a negative effect on patients' emotional, behavioral, and cognitive status. The pandemic adversely impacted clinicians financially, personally, and emotionally, with more than half (67%) reporting that they experienced burnout. This study found that the COVID-19 pandemic adversely impacted clinicians working in LTC settings, their patients' wellbeing, and the delivery of mental health services. Understanding the impact that the COVID-19 pandemic has had on LTC patients and clinicians alike has implications for the provision of services during future pandemics.

